# Development and Validation of a Machine Learning Model for Identifying Novel HIV Integrase Inhibitors

**DOI:** 10.7759/cureus.86326

**Published:** 2025-06-18

**Authors:** Blessed T Mukuhlani

**Affiliations:** 1 College of Medicine, University of Zimbabwe, Harare, ZWE

**Keywords:** drug discovery, hiv integrase inhibitors, logistic regression, machine learning, random forest

## Abstract

Human immunodeficiency virus (HIV) integrase inhibitors play a critical role in antiretroviral therapy, but the emergence of drug resistance necessitates the discovery of novel compounds. Machine learning (ML) offers a data-driven approach to accelerate drug discovery by predicting potential inhibitors with high efficacy. This study utilized a curated dataset of known HIV integrase inhibitors and employed feature engineering techniques to extract molecular descriptors. Random forest and logistic regression models were trained to classify compounds based on their inhibitory potential. Model performance was evaluated using accuracy, precision, recall, and the area under the receiver operating characteristic curve (AUC-ROC). The random forest model demonstrated superior predictive performance, achieving an AUC-ROC of 0.886, an accuracy of 0.815, and a precision of 0.79. Key molecular features, including hydrogen bond donors, rotatable bonds, and molecular weight, were identified as crucial determinants of inhibition. The models successfully screened novel compounds with high predicted inhibitory potential. Machine learning (ML) provides a powerful tool for the rapid identification of potential HIV integrase inhibitors. This study highlights the importance of molecular descriptors in predicting inhibitory activity and demonstrates the feasibility of ML-driven drug discovery. Future work will focus on refining model generalization, expanding datasets, and developing a user-friendly platform via Streamlit to enhance accessibility for researchers and drug developers.

## Introduction

More than 75 million people worldwide have been infected with the human immunodeficiency virus (HIV), and approximately 37 million individuals are currently living with the infection. If left untreated, HIV causes progressive CD4+ T cell depletion and widespread immunological dysfunction, which significantly increases the risk of opportunistic infections and malignancies [1]. Despite global preventive and therapeutic efforts, HIV remains a significant public health challenge, particularly in resource-limited settings [2]. In the United States, the epidemic shows marked geographical disparities, with the Southern region experiencing higher prevalence rates, especially in rural areas [3]. Meanwhile, countries like China have undertaken multi-decade comprehensive response efforts following domestic outbreaks among high-risk populations [4].

The introduction of combination antiretroviral therapy (cART), typically involving at least three active agents from different drug classes, has revolutionized HIV management by reducing morbidity, prolonging survival, and lowering transmission risk [5]. However, the complex multistep life cycle of HIV, including its ability to integrate its genome into host chromosomal DNA, remains a formidable barrier to eradication. This integration step, mediated by the HIV integrase (IN) enzyme, is not only essential for viral replication but also allows the virus to establish a latent reservoir [6].

Integrase strand transfer inhibitors (INSTIs) represent the most recent class of antiretroviral drugs approved for HIV treatment. These inhibitors, which include raltegravir (RAL), elvitegravir (EVG), and dolutegravir (DTG), act by blocking the integration of viral DNA into the host genome. While INSTIs have demonstrated high potency and a favorable resistance profile, their use is not without drawbacks. Neuropsychiatric side effects, notably with DTG, and metabolic concerns, such as weight gain, have been documented [7,8]. Furthermore, the rise of drug-resistant HIV strains, especially in treatment-experienced or pediatric populations, underscores the need for continued drug innovation [9].

Advances in structural biology and structure-activity relationship (SAR) analysis of known integrase inhibitors have revealed promising strategies for designing next-generation compounds [10]. Nevertheless, traditional drug discovery remains slow and resource-intensive. In recent years, machine learning (ML) has emerged as a transformative approach in pharmaceutical research, offering tools for pattern recognition, compound prioritization, and predictive modeling [11]. ML applications span all stages of drug discovery, from virtual screening and de novo drug design to toxicity prediction and pharmacophore modeling. Despite the promise, the application of ML to HIV integrase inhibitor discovery remains underexplored. A limited number of studies have rigorously validated ML models specific to this target, particularly using large, curated datasets and diverse algorithmic approaches such as support vector machines, random forests, and artificial neural networks. Furthermore, integrating ML with cheminformatics and virtual screening tools can improve decision-making in early drug discovery and reduce the risk of failure in downstream development stages [12-14].

Study aim

In light of these challenges and opportunities, this study aims to develop and validate a machine learning model capable of identifying novel HIV integrase inhibitors. By leveraging publicly available bioactivity data and applying robust ML algorithms, this study seeks to accelerate the early identification of lead compounds and contribute to the next generation of HIV therapeutics.

Objectives

The objectives of this study are: 1. To curate and pre-process a dataset of known HIV integrase inhibitors for machine learning-based analysis; 2. To extract and analyze molecular descriptors using feature engineering techniques relevant to integrase inhibition; 3. To develop and train machine learning models (random forest and logistic regression) to classify compounds based on their inhibitory potential against HIV integrase; 4. To evaluate the performance of the models using metrics such as accuracy, precision, recall, and AUC-ROC.

This article was previously posted to the Research Square preprint server on March 10, 2025.

## Materials and methods

Data acquisition and preparation

The primary data source for this study is the ChEMBL database [15], which provides extensive bioactivity data on various compounds, including their interactions with biological targets. The target protein of interest, HIV integrase, was identified using ChEMBL’s target search functionality. Compounds associated with measured IC50 values for HIV integrase inhibition were retrieved. To ensure a high-quality dataset, only experimentally verified data points were included, excluding computationally predicted or inferred values. This study employed a machine learning pipeline implemented in Python, leveraging the computational environment provided by Google Colab for model development and evaluation. A curated dataset of known HIV integrase inhibitors was sourced from publicly available databases.

Once retrieved, the dataset underwent a series of data-cleaning steps. Redundant columns, such as activity_properties, action_type, and ligand_efficiency, were removed to streamline the dataset. The SMILES (Simplified Molecular Input Line Entry System) strings, which represent chemical structures, were standardized using RDKit, ensuring uniform molecular representations. This step included removing salts, sanitizing structures, and normalizing tautomers to prevent duplicate entries. Since IC50 values (half-maximal inhibitory concentration) are often skewed, they were converted to pIC50 values (negative logarithm of IC50) for better model performance and improved data linearity. Any negative or biologically implausible IC50 values were filtered out. Additionally, missing values in critical columns, particularly pIC50, were handled by removing incomplete data points.

To further refine the dataset, exploratory data analysis (EDA) was conducted. The distributions of pIC50 values were visualized to confirm normality, and summary statistics were computed to assess variability. Correlation analysis was performed to identify potential relationships between molecular descriptors and pIC50 values, providing insights into the most influential features for model prediction. A heatmap was generated to visualize these correlations, highlighting significant interactions among descriptors.

Feature engineering

To enhance predictive performance, molecular descriptors were calculated from the standardized SMILES (Simplified Molecular Input Line Entry System) strings using RDKit’s Descriptors module. These descriptors capture important physicochemical properties of the molecules, which are critical for modeling drug-target interactions. The selected descriptors included: Molecular Weight (MW): Indicates molecular size and drug-likeness; LogP (Octanol-water partition coefficient): Measures hydrophobicity, which affects membrane permeability; Hydrogen Bond Donors (HBD) and Acceptors (HBA): Define molecular interactions with biological targets; Topological Polar Surface Area (TPSA): Represents molecular polarity, affecting solubility and bioavailability; Number of Rotatable Bonds: Reflects molecular flexibility, influencing binding affinity.

Feature selection was performed using recursive feature elimination (RFE) with a random forest classifier, ensuring that only the most relevant descriptors were retained. RFE iteratively removes less important features to optimize model performance. The final set of features was chosen based on importance scores, with the four most predictive descriptors used for training the machine learning models.

Model development and training

For the predictive modelling task, binary classification was implemented, where pIC50 values were categorized into inhibitors (1) and non-inhibitors (0) using a predefined threshold of 5. Compounds with pIC50 values greater than 5 were classified as inhibitors, while those below 5 were labeled as non-inhibitors.

The dataset was split into training (80%) and testing (20%) sets to ensure robust model evaluation. Two machine learning models were selected for comparison: 1. Random Forest Classifier: A widely used ensemble learning method known for handling high-dimensional data and reducing overfitting; 2. Logistic Regression: A baseline statistical model for binary classification, offering interpretability and computational efficiency.

To optimize performance, hyperparameter tuning was conducted using GridSearchCV, which systematically tests multiple parameter combinations. For random forest, hyperparameters such as the number of trees (100) and 2 minimum samples per split were optimized. The best-performing parameter set was selected based on cross-validation accuracy.

Model evaluation and performance metrics

Once trained, the models were evaluated on the testing dataset using multiple performance metrics: Accuracy: Measures overall correctness in classification; AUC-ROC (Area under the receiver operating characteristic curve): Assesses the ability to distinguish between inhibitors and non-inhibitors; Precision: Evaluates the proportion of correctly identified inhibitors; Recall: Measures the model’s ability to detect inhibitors without missing significant instances; F1-score: Balances precision and recall for a comprehensive performance measure.

Feature importance was also analyzed for the random forest model, identifying which molecular descriptors contributed most to the predictions. This information is valuable for understanding the molecular properties that drive HIV integrase inhibition.

## Results

The performance of the random classifier and logistic regression model was evaluated using accuracy, AUC-ROC, precision, recall, and F1-score. The results are summarized in Table 1.

**Table 1:** Comparison between random classifier and logistical regression AUC-ROC: area under the receiver operating characteristic curve; F1 score: harmonic mean of the precision

Metric	Random Classifier	Logistic Regression
Accuracy	0.816	0.58
AUC-ROC	0.886	0.595
Precision	0.792	0.571
Recall	0.79	0.187
F1-score	0.791	0.282

Model performance comparison

The random classifier outperformed logistic regression across all key metrics. The random classifier achieved an accuracy of 0.816 and an AUC-ROC of 0.886, while logistic regression achieved an accuracy of 0.580 and an AUC-ROC of 0.595. Additionally, logistic regression had a notably lower recall (0.187) and F1-score (0.282) compared to the random classifier (0.790 and 0.791, respectively), indicating challenges in correctly identifying positive instances.

Feature importance analysis revealed that topological polar surface area (TPSA) was the strongest predictor of inhibitory activity, followed by molecular weight (MW) and LogP (lipophilicity). Rotatable bonds showed lower importance, suggesting a lesser impact on classification. These results demonstrate the effectiveness of random forest in capturing the complex, non-linear relationships inherent in the data, highlighting the importance of molecular polarity, size, and lipophilicity in predicting inhibitory activity.

Table 2 highlights the feature importance analysis across logistic regression and random forest models, focusing on six molecular descriptors. In the logistic regression model, LogP had the highest negative coefficient at -0.15, suggesting an inverse relationship with the outcome, whereas HBD (0.065) and HBA (0.034) had positive but relatively small contributions. Rotatable bonds also showed a negative influence with a coefficient of -0.056, while TPSA and MW contributed minimally with coefficients of -0.006 and 0.002, respectively. In contrast, the random forest model assigned the highest importance to TPSA (0.242), closely followed by LogP (0.236) and MW (0.235), indicating these descriptors played a critical role in prediction. HBD, HBA, and rotatable bonds had lower importance values at 0.091, 0.09, and 0.11, respectively. Overall, the random forest model appears to distribute importance more evenly across descriptors, whereas logistic regression emphasizes fewer features with small coefficient magnitudes.

**Table 2:** The analysis of feature importance across logistic regression and random forest models TPSA: topological polar surface area; MW: molecular weight; HBA: hydrogen bond acceptors; HBD: hydrogen bond donors

Descriptor	Logistic Regression Coefficient	Random Forest Importance
LogP	-0.15	0.236
HBD	0.065	0.091
Rotatable Bonds	-0.056	0.11
HBA	0.034	0.09
TPSA	-0.006	0.242
MW	0.002	0.235

**Figure 1:**
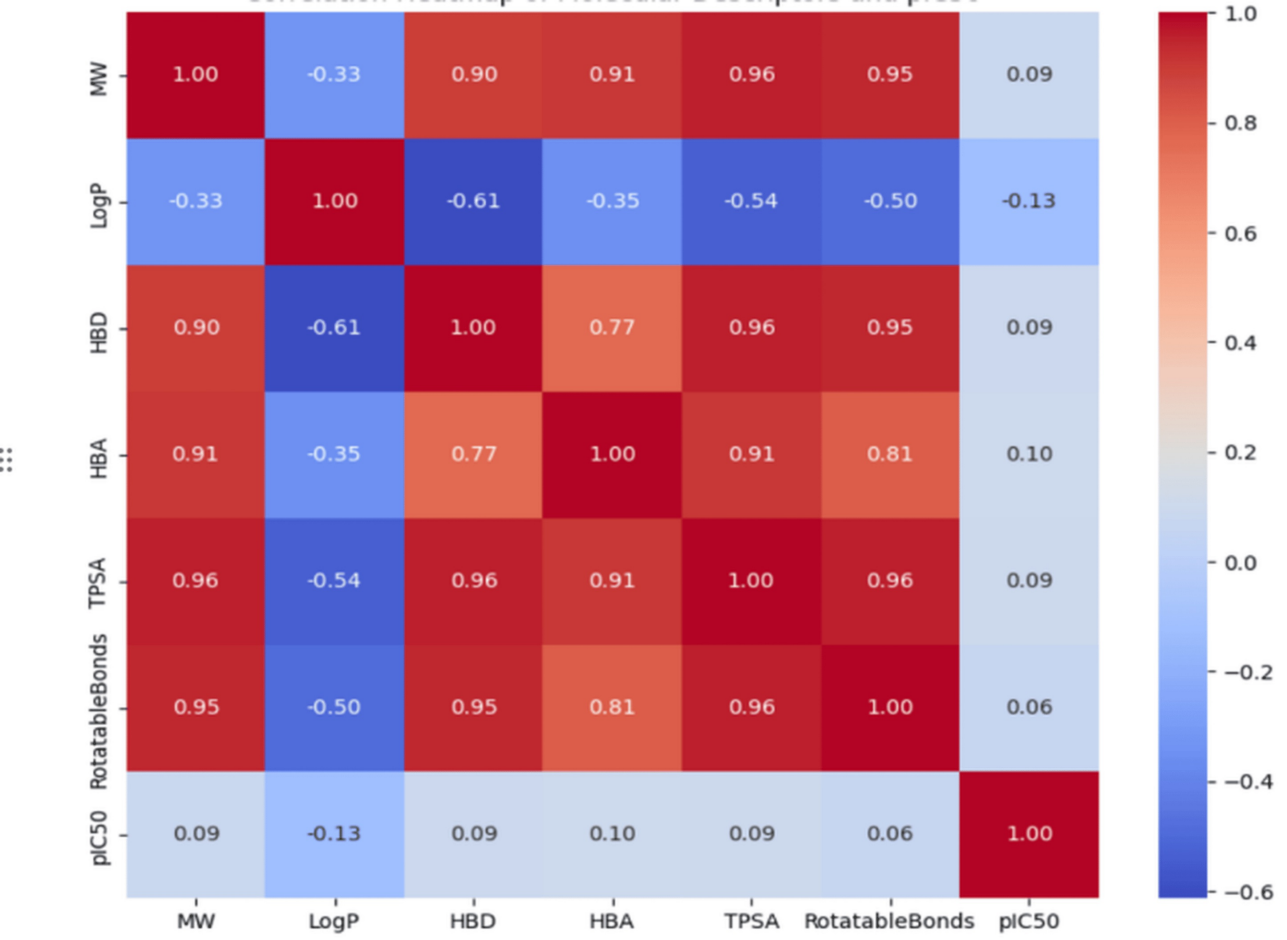
Correlation heatmap of molecular descriptors and pIC50 TPSA: topological polar surface area; MW: molecular weight; HBA: hydrogen bond acceptors; HBD: hydrogen bond donors

A correlation heatmap was generated to assess the relationships between molecular descriptors and pIC50 values (Figure 1). Most molecular descriptors - MW, HBD, HBA, TPSA, and the number of rotatable bonds - exhibited strong positive intercorrelations, with Pearson correlation coefficients > 0.90. This suggests potential multicollinearity among these descriptors, which may affect some machine learning models sensitive to correlated features (e.g., linear regression).

Interestingly, LogP showed a weak negative correlation with most other descriptors and pIC50 (r = -0.13), indicating it may provide orthogonal information relevant to modeling. However, none of the descriptors demonstrated a strong correlation with pIC50, with values hovering around ±0.10. This suggests that no single molecular descriptor alone is predictive of bioactivity, and supports the use of machine learning approaches to uncover complex, nonlinear relationships among features. These results justify the inclusion of multiple descriptors in the model, while also highlighting the need for dimensionality reduction techniques or regularization to mitigate redundancy.

**Figure 2:**
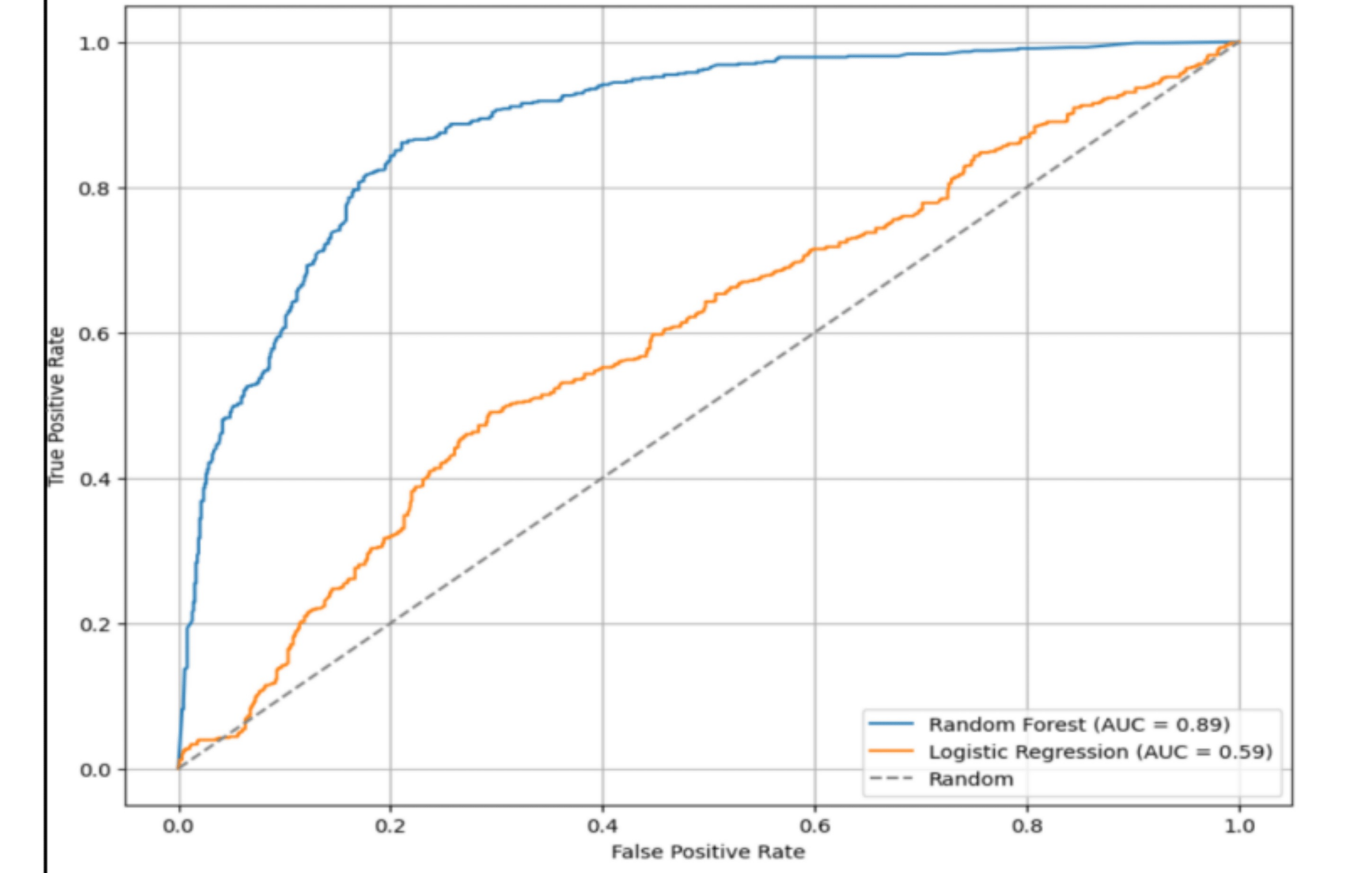
Receiver operating characteristic (ROC) curve comparing the random forest model against the logistic regression model

The ROC curve (Figure 2) comparison illustrates a clear performance advantage of the Random Forest model over Logistic Regression in classifying the dataset. The Random Forest achieves an AUC (Area Under the Curve) of 0.89, indicating excellent discriminatory power and a strong ability to distinguish between the two classes. In contrast, the Logistic Regression model yields a much lower AUC of 0.59, only slightly better than random guessing (AUC = 0.50). This disparity underscores the superior performance of ensemble methods like Random Forest, especially when dealing with complex, nonlinear relationships among features. The curve for Random Forest hugs the top-left corner of the plot, indicating a high true-positive rate and a low false-positive rate across various thresholds, which is crucial for reliable classification in predictive modeling.

The most active compound has a pIC50 value of 9.34 and a SMILES representation of:

O=C(NNC(=O)c1ccccc1)c1ccccc1.O=C(NNCC(O)COc1ccccc1)c1ccccc1

This compound also exhibits favorable physicochemical properties, including a LogP of 2.2391, 8 HBD, 9 HBA, a TPSA of 189.48 Å², and 9 rotatable bonds. These descriptors suggest that the compound maintains a balance between hydrophilicity and lipophilicity while also showing potential for strong biological interactions, justifying its selection for further experimental validation or in vitro testing.

## Discussion

The comparative performance evaluation between logistic regression and the random forest classifier highlights the superiority of ensemble learning methods in modeling complex molecular data. Random forest significantly outperformed logistic regression across all key metrics, including accuracy (0.816 vs. 0.580), AUC-ROC (0.886 vs. 0.595), precision (0.792 vs. 0.571), recall (0.790 vs. 0.187), and F1-score (0.791 vs. 0.282), emphasizing its robustness in handling non-linear feature interactions and imbalanced classification. The low recall and F1-score from logistic regression reflect its limitations in capturing the underlying patterns of bioactivity, potentially due to multicollinearity among descriptors or insufficient flexibility in modeling nonlinear relationships.

Feature importance analysis further reinforces these findings. While logistic regression assigns the highest negative coefficient to LogP, indicating an inverse linear relationship with the target, the random forest model identifies TPSA, MW, and LogP as the most influential descriptors. This contrast suggests that although some features may show minimal effect in linear models, they may play a significant role in nonlinear models like random forests, where interactions and thresholds are learned adaptively. Notably, LogP shows up as important in both models-albeit with opposing implications-highlighting its relevance but also the complexity of its relationship with biological activity.

The correlation analysis revealed strong inter-descriptor correlations, particularly among MW, TPSA, HBA, HBD, and rotatable bonds (r > 0.90), raising concerns of multicollinearity. However, none of these descriptors exhibited a strong direct correlation with pIC50, supporting the need for advanced machine learning techniques that can model intricate, non-obvious relationships. The low correlation between individual features and pIC50 further justifies the use of multivariate models and the potential benefit of applying dimensionality reduction or regularization strategies in future work.

The ROC curve analysis clearly demonstrates the discriminative advantage of the random forest model. The AUC of 0.89 suggests excellent performance in distinguishing active from inactive compounds, compared to an AUC of 0.59 for logistic regression, barely above the chance level. The steep rise of the random forest ROC curve near the y-axis indicates a high true positive rate at low false-positive rates, making it especially valuable for predictive tasks in drug discovery, where false negatives could result in overlooking potent candidates.

The identification of the most active compound, with a pIC50 of 9.34, further validates the modeling approach. This molecule possesses a favorable LogP (2.2391), optimal hydrogen bonding profile (8 HBD and 9 HBA), and high TPSA (189.48 Å²), indicating a strong potential for solubility and bioavailability. Its structural features suggest promising lead-likeness and a balanced hydrophilic-lipophilic profile, meriting further investigation in experimental settings.

The present study developed a random forest model using a comprehensive set of 204 molecular descriptors to classify compounds as active or inactive against HIV-1 integrase. This approach showed high accuracy, with robust internal validation and insights into feature importance. When compared to previous studies, several distinctions and similarities can be observed.

Parvez et al. employed multiclass classification using support vector machines (SVM) and optimized their model using the Boruta feature selection algorithm. They reported slightly improved performance with selected features and used their validated models for virtual screening against the ChemBridge library. Notably, compounds predicted to be highly active formed stable interactions with key catalytic residues (Asp64, Glu152, and Asn155), which aligns with findings from our docking study, underscoring the relevance of targeting these residues for HIV-integrase inhibition [16].

Machado et al. addressed class imbalance issues and conducted hyperparameter tuning across various machine learning algorithms to screen natural compounds from the Natural Product Atlas. While their focus was on ligand-based screening and applicability domains to distinguish “predictable” versus “unknown” chemical space, our study prioritized descriptor-based modeling and molecular docking. Nevertheless, both studies highlight the utility of ML to narrow down candidate compounds from large libraries efficiently [17].

Zhou et al. integrated both molecular descriptors and fingerprints to improve predictive accuracy using recursive partitioning (RP) and Naive Bayes (NB) classifiers. Their models showed high classification accuracy (up to 88.3%) and revealed key molecular fragments responsible for bioactivity. Additionally, they employed CoMFA and CoMSIA techniques to explore structure-activity relationships. Unlike our random forest model, which emphasizes ensemble learning and descriptor importance, their approach relied more on molecular fingerprints and QSAR modeling to derive insights for lead optimization [18].

In summary, the results support the adoption of ensemble methods, such as random forest, for predictive modeling in cheminformatics. These approaches not only outperform traditional linear models in terms of predictive power but also provide richer insights into feature importance and compound prioritization. Future directions may include incorporating additional molecular descriptors, using deep learning architectures, and applying external validation on independent datasets to further enhance model generalizability and reliability.

Limitations

Despite the promising performance of the random forest model and insightful feature analysis, several limitations should be acknowledged. First, the dataset size and diversity may limit the generalizability of the findings. If the compounds are structurally similar or biased toward specific chemical classes, the model may not perform as well on external or more diverse datasets. Second, the strong intercorrelations among molecular descriptors suggest potential multicollinearity, which could affect the interpretability and stability of models like logistic regression. While ensemble methods are less sensitive to such redundancy, the presence of highly correlated features may still reduce model efficiency and inflate feature importance scores.

Moreover, the models were trained and validated on the same dataset without the use of an independent test set or external validation, which raises concerns about overfitting and the real-world predictive power of the models. Additionally, the pIC50 values were treated as a binary classification problem, potentially oversimplifying the underlying continuous bioactivity spectrum and discarding nuanced activity differences between compounds.

Lastly, the study did not consider advanced feature engineering or hyperparameter optimization, which could further enhance model performance. Techniques such as principal component analysis (PCA), RFE, or regularization methods (e.g., LASSO) were not applied and may be valuable in future iterations. Experimental validation of the top predicted compounds was also beyond the scope of this work, though it is essential to confirm biological relevance.

## Conclusions

The development of HIV integrase inhibitors remains a crucial aspect of antiretroviral therapy, particularly in the face of emerging drug resistance. This study highlights the potential of machine learning in accelerating drug discovery by identifying novel HIV integrase inhibitors with improved efficacy. By leveraging molecular descriptors and predictive modeling, our approach offers a data-driven framework for screening promising compounds, reducing both the time and cost associated with traditional drug discovery methods. The integration of random forest and logistic regression models demonstrated robust predictive performance, with key molecular features identified as critical determinants of inhibition.

## References

[1] Deeks SG, Overbaugh J, Phillips A, Buchbinder S (2015). HIV infection. Nat Rev Dis Primers.

[2] Obeagu EI, Obeagu GU (2022). An update on survival of people living with HIV in Nigeria. Internet.

[3] Sullivan PS, Johnson AS, Pembleton ES (2021). Epidemiology of HIV in the USA: epidemic burden, inequities, contexts, and responses. Lancet.

[4] He N (2021). Research progress in the epidemiology of HIV/AIDS in China. China CDC Weekly.

[5] Cihlar T, Fordyce M (2016). Current status and prospects of HIV treatment. Curr Opin Virol.

[6] Trivedi J, Mahajan D, Jaffe RJ, Acharya A, Mitra D, Byrareddy SN (2020). Recent advances in the development of integrase inhibitors for HIV treatment. Curr HIV/AIDS Rep.

[7] Max B (2019). Update on HIV integrase inhibitors for the treatment of HIV-1 infection. Future Virol.

[8] Anstett K, Brenner B, Mesplede T, Wainberg MA (2017). HIV drug resistance against strand transfer integrase inhibitors. Retrovirology.

[9] Dehority W, Abadi J, Wiznia A, Viani RM (2015). Use of integrase inhibitors in HIV-infected children and adolescents. Drugs.

[10] Rathbun RC, Lockhart SM, Miller MM, Liedtke MD (2014). Dolutegravir, a second-generation integrase inhibitor for the treatment of HIV-1 infection. Ann Pharmacother.

[11] Vamathevan J, Clark D, Czodrowski P (2019). Applications of machine learning in drug discovery and development. Nat Rev Drug Discov.

[12] Dara S, Dhamercherla S, Jadav SS, Babu CM, Ahsan MJ (2022). Machine learning in drug discovery: a review. Artif Intell Rev.

[13] Lima AN, Philot EA, Trossini GH, Scott LP, Maltarollo VG, Honorio KM (2016). Use of machine learning approaches for novel drug discovery. Expert Opin Drug Discov.

[14] Machado LA, Krempser E, Guimarães ACR (2022). A machine learning-based virtual screening for natural compounds capable of inhibiting the HIV-1 integrase. Front Drug Discov.

[15] (n.d). HIV integrase. http://www.ebi.ac.uk/chembl/search_results/hiv%20intergase.

[16] Parvez MK, Al-Dosari MS, Sinha GP (2022). Machine learning-based predictive models for identifying high active compounds against HIV-1 integrase. SAR QSAR Environ Res.

[17] Tunc H, Sari M, Kotil S (2023). Machine learning aided multiscale modelling of the HIV-1 infection in the presence of NRTI therapy. PeerJ.

[18] Zhou J, Hao J, Peng L (2021). Classification and design of HIV-1 integrase inhibitors based on machine learning. Comput Math Methods Med.

